# Investigation of Glucose–Water Mixtures as a Function of Concentration and Temperature by Infrared Spectroscopy

**DOI:** 10.3390/ijms24032564

**Published:** 2023-01-29

**Authors:** Maria Teresa Caccamo, Salvatore Magazù

**Affiliations:** 1 Dipartimento di Scienze Matematiche e Informatiche, Scienze Fisiche e Scienze della Terra, Università di Messina, Viale F.S. D’Alcontres, 31, 98166 Messina, Italy; 2 Consorzio Interuniversitario Scienze Fisiche Applicate (CISFA), Viale F.S. D’Alcontres, 31, 98166 Messina, Italy

**Keywords:** glucose, Fourier Transform InfraRed, spectral distance, temperature

## Abstract

The main aim of the present paper is to characterize the hydration properties of glucose and the hydrogen bond network in glucose–water mixtures. For these purposes, temperature scans on ten concentration values of glucose–water mixtures were performed by means of Fourier Transform InfraRed (FTIR) spectroscopy. More specifically, in order to get this information an analysis of the intramolecular OH stretching mode, investigating the 3000–3700 cm^−1^ spectral range, was performed by means of an innovative approach based on the evaluation of the Spectral Distance (SD). The adopted procedure allows evaluating the glucose hydration number as well as characterizing the temperature behavior of the hydrogen bond network in the glucose–water mixtures. The obtained results for the hydration number are in excellent agreement with literature data and suggest the existence of a particular concentration value for which the hydrogen bond network shows a maximum strength.

## 1. Introduction

Glucose is an aldohexose, the main representative of oses (sugars), and it is also known as dextrose in its crystallized form. By convention, it is symbolized by Glc. It comes in the form of a white powder, with a sweet taste, and caramelizes at about 150 °C. It is soluble in water, ethanol and pyridine but insoluble in diethyl ether and many other organic solvents. In 1838, a committee of the Academy of Sciences decided to call the sugar present in grapes, in starch and in honey ‘glucose’, after the Greek term ‘gleukos’, which means sweet wine [1,2]. Émile Littré furnished another etymology with the adjective glukus (sweet taste), with two upsilons (Greek i), and the usual prefix became glyc, as in glycemia, glycogen, etc. It is a carbohydrate compound, consisting of six carbon atoms and an aldehyde group, referred to as aldohexose; it is an isomer of other sugars, in particular of mannose or fructose, with the formula C_6_H_12_O_6_ [3,4].

Water is one of the most abundant and essential molecules for life on Earth. About 60–70% of the human body is made up of water. Without it, life, as we know, would simply not exist. One of the important properties of water is that it is made up of polar molecules: the hydrogen and oxygen contained in water molecules (H_2_O) form polar covalent bonds. Water has many essential functions; in particular it is nourishment for all living beings because, for example, it is the building material of cells and a means of transporting carbohydrates and proteins to all parts of the body [5,6,7].

**Figure 1:**
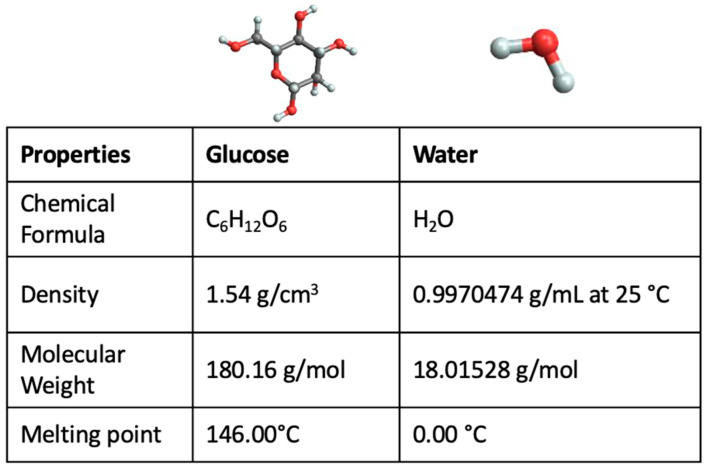
Properties of pure glucose and pure water together with their molecular structures.

Figure 1 reports some properties of pure glucose and water together with their molecular structure.

In the present paper we report the findings of an experimental study on the interaction mechanisms between glucose and water, with a specific focus on the hydration properties of glucose and on the hydrogen bond network in the glucose-water mixtures [8,9,10,11,12,13]. The study was carried out by means of infrared spectroscopy measurements performed in the middle infrared region, which covers the spectral range from 400 cm^−1^ to 4000 cm^−1^. This powerful and sensitive spectroscopic technique is essentially based on the molecular absorption of radiation in the infrared region of the electromagnetic spectrum and in the conversion of the absorption into molecular vibration, the absorption corresponding to the bonds present in the molecule [14,15,16,17,18,19,20,21].

Concerning the employed analysis methods, to get information on the hydrogen bond network of water molecules around the glucose molecule as well as to characterize the hydrogen bond network in the glucose-water mixtures, an innovative approach, based on the evaluation of the Spectral Distance, has been utilized [22,23,24].

The major goal of the present study is to establish a procedure for analyzing spectra of material systems composed of many components. In particular, due to the multicomponent nature of the investigated systems, a spectral analysis on the whole MID-Infrared region was performed, focusing on the spectral feature changes.

We analyzed the pure systems and ten concentration values for the glucose–water mixtures and ten different temperature values in the range of 20–50 °C for all the glucose–water mixtures values, focusing on the intramolecular OH stretching contribution, which encompasses the spectral range of 3000–3700 cm^−1^.

## 2. Results and Discussion

**Figure 2:**
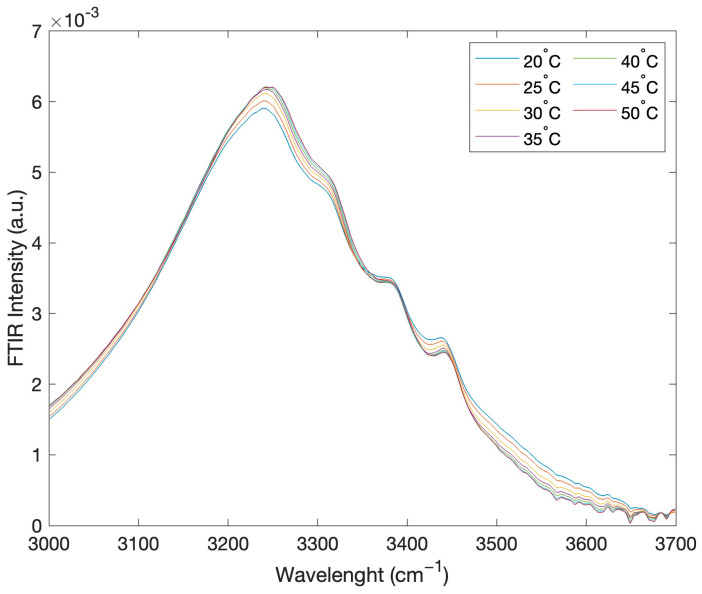
FTIR intramolecular OH stretching band intensity for the concentration value of 90% glucose and 10% water, as a function of temperature.

Figure 2 reports, as an example, the intramolecular OH stretching band intensity of the registered FTIR spectra, in the range 3000–3700 cm^−1^, for the concentration of 90% of glucose and 10% water, as a function of temperature in the range 20–50 °C.

**Figure 3:**
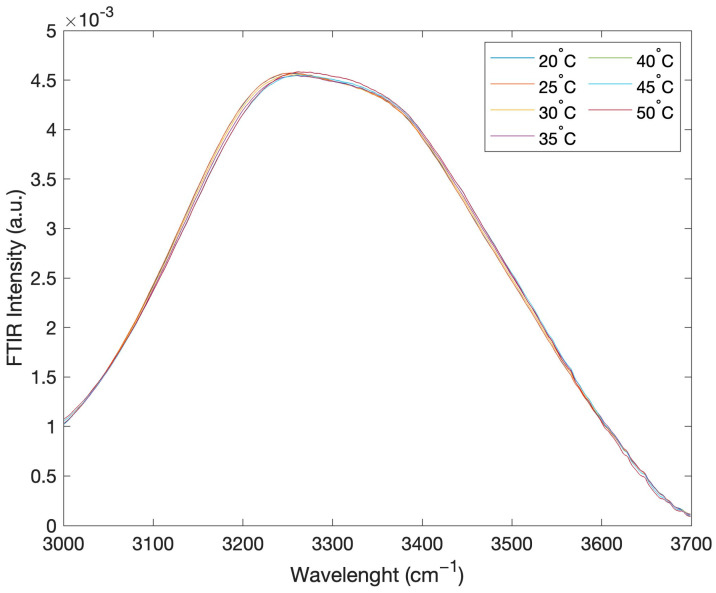
FTIR intramolecular OH stretching band for the concentration value of 50% glucose and 50% water, as a function of temperature.

Figure 3 reports the intramolecular OH stretching band intensity of the registered FTIR spectra, for the concentration of 50% glucose and 50% water, as a function of temperature.

As can be seen, these IR spectral contributions show relatively small differences with temperature but dramatically change in shape when the concentration value is changed.

**Figure 4:**
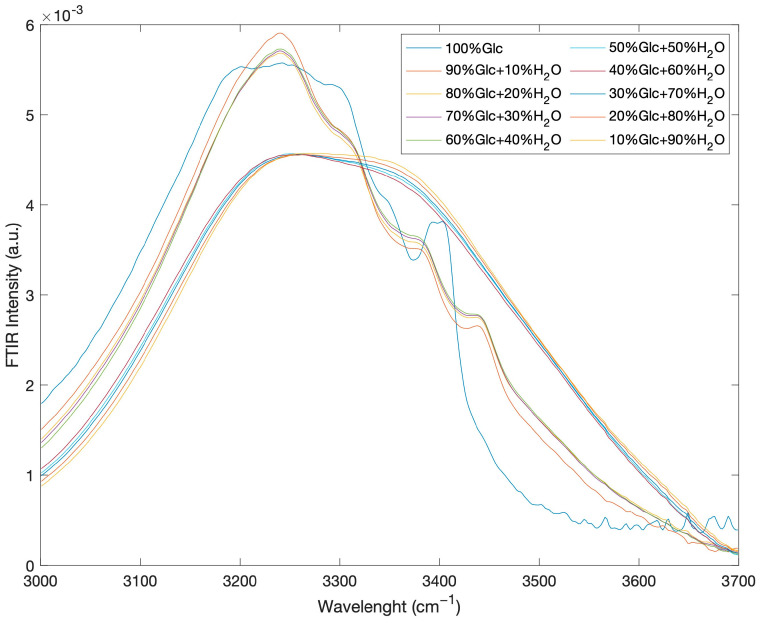
FTIR intramolecular OH stretching band as a function of concentration for glucose-water mixtures at the temperature value of *T* = 20 °C.

In order to better evidence the spectral changes when the intensive parameter concentration varies, Figure 4 reports the FTIR intramolecular OH stretching band, confined to the 3000–3700 cm^−1^ spectral range, as a function of concentration for the glucose-water mixtures at the temperature value of *T* = 20 °C.

The behaviour of the spectra profiles as a function of concentration shows that, starting from pure glucose and adding water, the shape spectral features show a clear transition around a concentration value corresponding to 50% glucose and 50% water.

**Figure 5:**
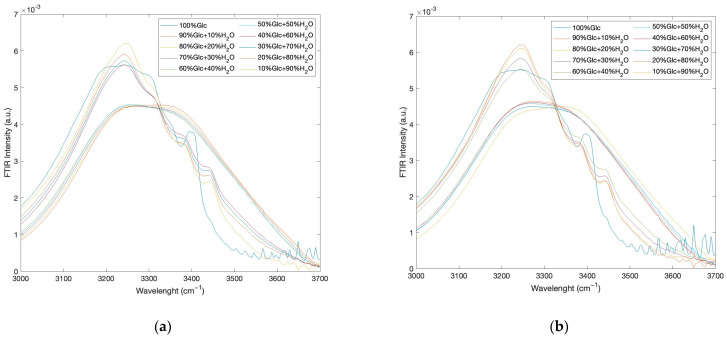
(**a**) Registered FTIR intramolecular OH stretching band intensities, as a function of concentration at the temperature value of *T* = 35 °C; (**b**) Registered FTIR intramolecular OH stretching band intensities, as a function of concentration at the temperature value of *T* = 50 °C.

In order to show that the same behaviour is also present at the other investigated temperatures, as an example, Figure 5a reports the registered FTIR intramolecular OH stretching band intensities, as a function of concentration at the temperature value of *T* = 35 °C while Figure 5b shows the registered FTIR intramolecular OH stretching band intensities, as a function of concentration at the temperature value of *T* = 50 °C.

As can be seen, the behaviour of the spectra profiles as a function of concentration shows that, independently of temperature, starting from pure glucose and adding water, the shape spectral features show a clear transition around a concentration value corresponding to 50% glucose and 50% water. Such a result reveals that the hydrogen bond network of the glucose–water mixture induces strong hydrogen bonds and influences the tetrahedral structure of the water molecules.

In order to extract quantitative information, an approach based on the Spectral Distance (SD) evaluation was employed.

More specifically, first we calculated the values of SD as a function of temperature, taking as reference the spectra registered at the lowest temperatures by means of Equation (1):

(1) $${SD}_{T}=\sqrt{{\left[I\left(\omega ,T\right)-I(\omega ,T_{r})\right]}^{2}\cdot \Delta \omega }$$

where $I\left(\omega ,T\right)$ is the intensity at the frequency *ω* and at a given temperature value T, $I\left(\omega ,T_{r}\right)$ is the intensity at the frequency *ω* and at a reference temperature value $T_{r}$, that in our case is $T_{r}=$ 20 °C, and Δ*ω* is the instrument frequency resolution. This procedure is revealed to be effective when, due to the overlapping of the vibrational modes it is difficult to assign the single spectral contributions.

**Figure 6:**
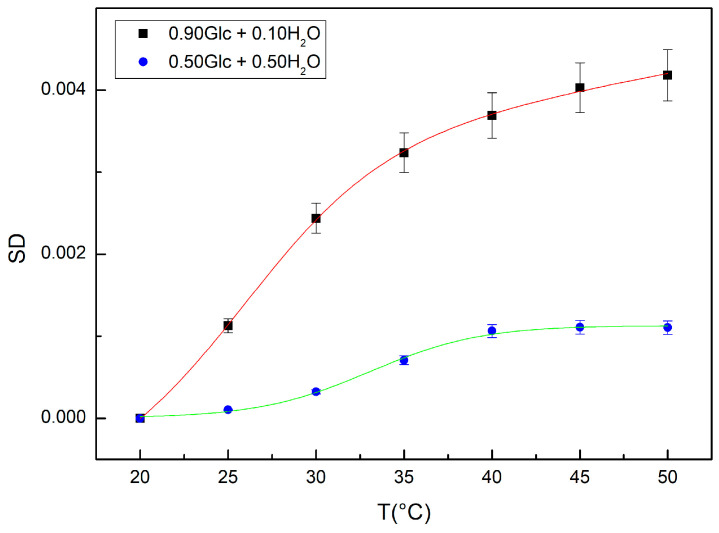
Values of SD calculated for the concentration of 90% glucose and 10% water (black square) together with the model fit (red curve) and values of SD calculated for the concentration of 50% glucose and 10% water (blue circle) together with the model fit (green curve).

Figure 6 reports a comparison between the values of SD calculated for the concentration value corresponding to 90% glucose and 10% water and for the concentration value corresponding to 50% glucose and 50% water. In the same figure, the fit results obtained by means of Equation (2) are reported; in particular, $T_{0}$ is the temperature inflection point and $A_{T}$ is the relaxation amplitude of the curve whose inverse value is connected to the system thermal restraint; $B_{T}$ represents the sigmoid steepness, and $C_{T}-D_{T}T$ is a linear fitting contribution [25,26,27]:

(2) $$SD(T)=A_{T}\left(1-\frac{1}{1+e^{B_{T}(T-T_{0})}}\right)+(C_{T}-D_{T}T)$$

From this analysis it emerges that for the concentration of 50% glucose and 50% water one registers a temperature inflection point at a value of $T_{0}$ = 33.06 °C with a value of amplitude $A_{T}$ equal to 0.00118, which are higher in respect to the values registered for the concentration of 90% glucose and 10% water, the values of which are $T_{0}$ = 25.97 °C and $A_{T}$ = 0.00375.

Such results show that at the concentration of 50% glucose and 50% water, the bond hydrogen network is stronger in respect to that corresponding to the concentration of 90% glucose and 10% water.

Concerning the spectra behavior as a function of the concentration, for evaluating the spectral distance, i.e., the SD, we used the following expression:

(3) $${SD}_{C}=\sqrt{\sum {\left[I\left(\omega ,c\right)-I(\omega ,c_{r})\right]}^{2}\cdot \Delta \omega }$$

in this case, I(ω,c) represents the intensity at the frequency ω at the concentration *c* while $I\left(\omega ,c_{r}\right)\;\mathrm{represents}\;\mathrm{the}\;\mathrm{intensity}\;\mathrm{at}\;\mathrm{the}\;\mathrm{frequency}\;\omega \;\mathrm{at}\;a\;\mathrm{reference}\;\mathrm{concentration}\;c_{r}\;=\;10\%$.

**Figure 7:**
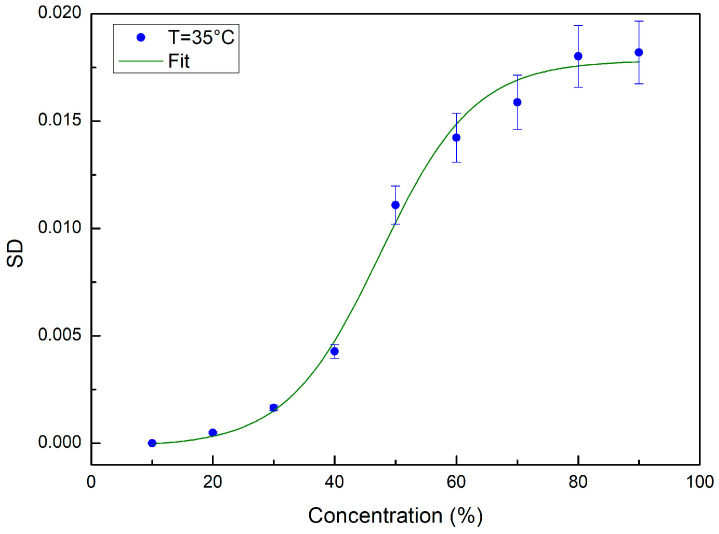
SD values calculated for *T* = 35 °C (blue circles) together with the fitting curve, i.e., green curve.

Figure 7 reports, as an example, the values of SD calculated, by means of Equation (3), for the temperature value of *T* = 35 °C. In this case as well, once the SD values were obtained, we used Equation (4) for fitting the evaluated SD data:

(4) $$SD(c)=A_{c}(1-\frac{1}{1+e^{B_{c}(c-c_{0})}})+(C_{c}-D_{c}c)$$

where $c_{0}$ is the concentration value corresponding to the inflection point, $A_{c}$ is the relaxation amplitude of the curve whose inverse value is connected to the system thermal restraint, $B_{c}$ represents the sigmoid steepness, and $C_{c}-D_{c}T$ is a linear fitting contribution.

From the data fitting procedure, we obtained a concentration transition value at c=47.49 for T= 35 °C.

The behavior of the SD values as a function of concentration confirms that, independently of temperature, starting from pure glucose and adding water, the shape spectral features show a clear transition around a concentration value corresponding to 50% glucose and 50% water. Such a result reveals that the hydrogen bond network of the glucose–water mixture induces strong hydrogen bonds and influences the tetrahedral structure of water molecules [28,29,30,31,32,33].

It is well known that one way to deconvolve the intramolecular OH stretching contribution into sub-bands makes reference to a partition of all the existing O-H bonds into O-H bonds belonging to tetrahedral or “open” arrangements and O-H bonds belonging to non-tetrahedral or “closed” arrangements. On these grounds, the registered shape transition of the intramolecular OH contribution by increasing the water content signals that the water molecules, at low water content, bind themselves to glucose, and hence contribute to the closed OH stretching sub-band, while, after reaching its full hydration, giving rise to water tetrahedral arrangements and so contributing to the open OH stretching sub-band [34,35].

In particular, at the concentration value corresponding to 50% glucose and 50% water, the bond hydrogen network is stronger in respect to all the other investigated concentration values and is less sensitive to temperature changes. These results furnish a glucose-hydration number value of 5 and suggest that at such a concentration value the glucose–water systems constitute more stable dynamical structures.

## 3. Materials and Methods

D(+)-glucose monohydrate and distilled water were purchased from Aldrich-Chemie. Infrared data were collected in the temperature range of 20–50 °C; the investigated concentration values, expressed as weight fractions, i.e., (grams of Glc)/(grams of Glc + grams of H_2_O) were: 0.0; 0.10; 0.20; 0.30; 0.40; 0.50; 0.60; 0.70; 0.80; 0.90. The samples were prepared using a magnetic stirrer.

The FTIR absorption spectra were acquired by means of a Vertex 70 V spectrometer from Bruker Optics using a Platinum diamond ATR, in which the infrared light passes through a crystal of diamond, and then through the sample, which is pressed onto this crystal. The spectra were collected in the middle spectral range of 400–4000 cm^−1^; the total data points in the specific spectral regions were 1866. Each spectrum was averaged over 64 scans to achieve an acceptable S/N ratio. Before proceeding to the interpretation of the obtained spectra, data pre-processing was applied employing using the Bruker OPUS/Mentor software and the MATLAB environment.

## 4. Conclusions

In this work the results obtained by means of FTIR spectroscopy measurements carried out as a function of temperature for ten concentration values of glucose–water mixtures are presented. More specifically, an analysis of the intramolecular OH stretching mode, investigating the 3000–3700 cm^−1^ spectral range, was performed by means of an approach based on the evaluation of the SD. The adopted procedure allowed us to evaluate a hydration number for glucose of five and to characterize the temperature behavior of the hydrogen bond network in the glucose–water mixtures. The obtained results for the hydration number are in excellent agreement with literature data and suggest the existence of a particular concentration value, corresponding to 50% glucose and 50% water, for which the hydrogen bond network shows a maximum strength.
